# Self-amplified photo-induced gap quenching in a correlated electron material

**DOI:** 10.1038/ncomms12902

**Published:** 2016-10-04

**Authors:** S. Mathias, S. Eich, J. Urbancic, S. Michael, A. V. Carr, S. Emmerich, A. Stange, T. Popmintchev, T. Rohwer, M. Wiesenmayer, A. Ruffing, S. Jakobs, S. Hellmann, P. Matyba, C. Chen, L. Kipp, M. Bauer, H. C. Kapteyn, H. C. Schneider, K. Rossnagel, M. M. Murnane, M. Aeschlimann

**Affiliations:** 1 I. Physikalisches Institut, Georg-August-Universität Göttingen, 37077 Göttingen, Germany; 2 Department of Physics and Research Center OPTIMAS, University of Kaiserslautern, 67663 Kaiserslautern, Germany; 3 JILA, University of Colorado and NIST, Boulder, Colorado 80309-0440, USA; 4 Institute of Experimental and Applied Physics, University of Kiel, 24098 Kiel, Germany; 5 Department of Physics, Massachusetts Institute of Technology, Cambridge, Massachusetts 02139, USA; 6 Francis Bitter Magnet Laboratory, Massachusetts Institute of Technology, Cambridge, Massachusetts 02139, USA

## Abstract

Capturing the dynamic electronic band structure of a correlated material presents a powerful capability for uncovering the complex couplings between the electronic and structural degrees of freedom. When combined with ultrafast laser excitation, new phases of matter can result, since far-from-equilibrium excited states are instantaneously populated. Here, we elucidate a general relation between ultrafast non-equilibrium electron dynamics and the size of the characteristic energy gap in a correlated electron material. We show that carrier multiplication via impact ionization can be one of the most important processes in a gapped material, and that the speed of carrier multiplication critically depends on the size of the energy gap. In the case of the charge-density wave material 1*T*-TiSe_2_, our data indicate that carrier multiplication and gap dynamics mutually amplify each other, which explains—on a microscopic level—the extremely fast response of this material to ultrafast optical excitation.

## Abstract (web summary)

The non-equilibrium dynamics of correlated electron materials are still poorly understood. Here, the authors use time- and angle-resolved photoemission spectroscopy to show that carrier multiplication is important in initial non-equilibrium dynamics of 1*T*-TiSe_2_ and depends on the size of the energy gap.

## Graphical abstract

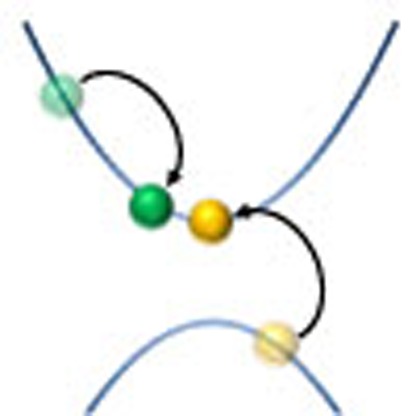

In recent years, femtosecond time-domain spectroscopy has emerged as an important tool for revealing the character of the dominant interactions in correlated electron materials^1^,^2^,^3^,^4^,^5^,^6^,^7^,^8^,^9^,^10^,^11^,^12^. In such experiments, after impulsive excitation by an ultrafast laser pulse, the temporal evolution of selected order parameters can be mapped using optical, X-ray and electron spectroscopies. These data can reveal the characteristic timescales of the relaxation in the material, and help to identify the underlying couplings and dominant interactions^13^. A critical assumption in interpreting these data is that transient non-equilibrium states can be compared with states of the system in thermal equilibrium. However, this assumption is only seldom justified, in particular on very short timescales, when pronounced non-equilibrium distributions of photoexcited carriers are present. Moreover, little or no attention has been paid to uncovering far-from-equilibrium responses of materials that might be common to all strongly excited correlated electron materials. For example, if there are universal relations between the non-equilibrium electron relaxation dynamics and the size of the characteristic energy gaps in a correlated material.

To address this challenge, here we use time- and angle-resolved photoemission (trARPES) to study the charge-density wave (CDW) system 1*T*-TiSe_2_. At liquid nitrogen temperature, this material shows a CDW gap of ∼100 meV^14^, which is known to get quenched on femtosecond timescales after intense ultrafast optical excitation^3^,^13^,^15^,^16^. Moreover, the speed and magnitude of the quenching of the CDW phase in 1*T*-TiSe_2_ strongly depends on excitation fluence, and thus can easily be adjusted in our experiments. By analysing the fast electron dynamics far away from equilibrium, we identify the dominant role in the initial response of the material played by energy-gap-dependent carrier multiplication via impact ionization. Moreover, because the CDW gap size in 1*T*-TiSe_2_ is reduced on femtosecond timescales, there is a mutually induced speed-up of carrier multiplication and CDW quenching. This coupled response explains—on a microscopic level—the extremely fast response of 1*T*-TiSe_2_ to femtosecond laser excitation.

## Results

### Time-resolved photoemission spectroscopy

**Figure 1:**
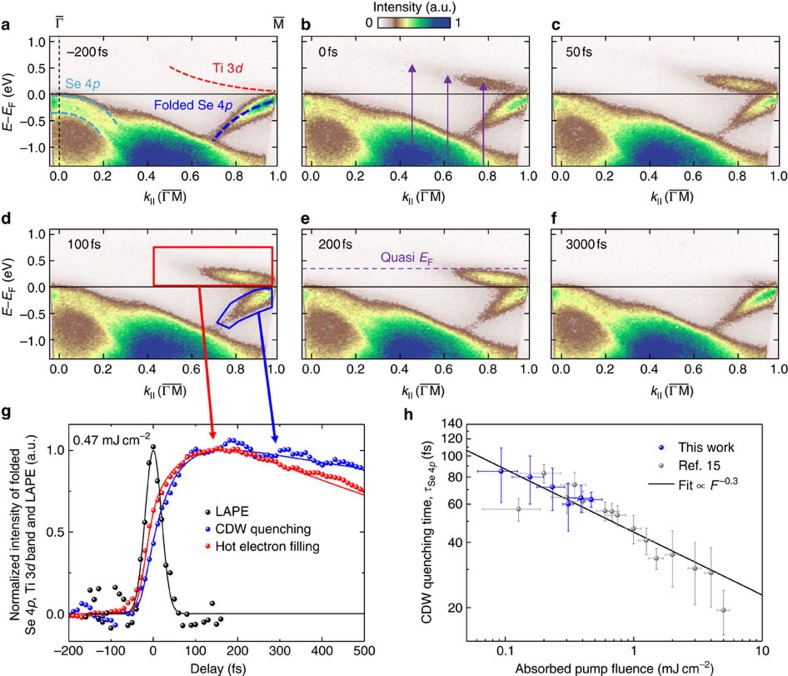
Spectra of the transient electronic dynamics of photoexcited 1*T*-TiSe_2_. (**a**–**f**) Photoemission maps of the electronic response of the backfolded Se 4*p* states (blue area), and the hot-electron dynamics in the Ti 3*d* band (red area) at different times. The absorbed fluence of the 1.6 eV, 32 fs, *p*-polarized pump pulses was 0.47 mJ cm^−2^. The polarization of the 22 eV XUV pulses was *p*. (**g**) Suppression of the spectral weight of the backfolded Se 4*p* states (blue data points and line), which is indicative of the quenching of the CDW. This curve is obtained by mirroring the spectral-weight dynamics at the *x* axis, so that the timescales can be compared with the electron accumulation in the Ti 3*d* band (red data points and line). The black data and line shows the cross-correlation from pump and probe pulse, extracted from the laser-assisted photoelectric effect, LAPE^32^ (Supplementary Fig. 1). (**h**) Summary of the extracted CDW quenching times *τ*_Se 4*p*_ as a function of absorbed pump fluence in comparison to our previous data set^15^. The fit curves for the extraction of *τ*_Se 4*p*_ are shown in Fig. 4c as lines. The error bars for *τ*_Se 4*p*_ are obtained from the fits, while the error bars of the absorbed pump fluence originate from the measuring inaccuracy of average power and spot size of the pump pulse.

In our experiments, we excite the CDW insulator 1*T*-TiSe_2_ using 32 fs, 1.6 eV infrared pulses. The transient response of the material is then monitored using extreme-ultraviolet trARPES with high spectral (<150 meV) and high temporal (Δ*τ*_probe_≈33 fs) resolution, as well as high extreme ultraviolet (XUV) photon flux^17^,^18^. This allows us to directly capture transient changes in the band structure and occupation on femtosecond timescales, and to separate the fast non-equilibrium carrier dynamics from evolution of the electronic response on a longer timescale (>200 fs). Figure 1a shows a photoemission spectrum of 1*T*-TiSe_2_ in the CDW phase for a pump–probe delay of *t*=−200 fs (that is, below the transition temperature of *T*=200 K and before optical excitation). In the CDW phase, the real-space unit cell doubles its size in all three directions compared with the normal high-temperature phase, with the size of the Brillouin zone correspondingly halved. The 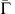 and 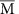 points in the normal phase become equivalent 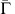 points in the CDW phase, that is, the hole-like Se 4*p* bands at the original 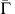 point are backfolded to the equivalent 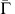 point in the CDW phase (marked with folded Se 4*p*). The spectral weight of the backfolded Se 4*p* bands (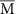 point) is the electronic fingerprint of the CDW phase that we can follow in our time-resolved experiment^13^,^14^,^15^,^19^. Furthermore, during the CDW transition via cooling of the sample, the Se 4*p* bands shift to larger binding energies (60 meV shift from room temperature to 100 K at the 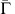 point^20^) and a gap opens between the occupied Se 4*p* and the unoccupied Ti 3*d* bands (Δ*E*_gap_≈100 meV at liquid nitrogen temperature^14^).

The series of photoemission maps shown in Fig. 1a–f correspond to time-resolved snapshots of the electronic quasi-particle states after excitation with a 1.6 eV, 32 fs, *p*-polarized infrared pulse. Upon excitation, the previously unoccupied Ti 3*d* band, marked by a red dashed line and Ti 3*d* in Fig. 1a, is filled with electrons originating from the Se 4*p*_x,y_ bands that are nearly parallel and 1.6 eV below the Ti 3*d* band in the second half of the Brillouin zone in 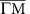 direction^15^,^21^,^22^ (c.f. purple arrows in the 0 fs snapshot, Fig. 1b). The strong optical Se 4*p*_x,y_ to Ti 3*d* excitation induces an initial non-Fermi-Dirac redistribution of highly excited electrons in the Ti 3*d* band, which then migrate from the extended excitation region to the Ti 3*d* conduction band minimum (200 fs snapshot, Fig. 1e). Our main observation here is that beyond the initial optical excitation (temporal width of the pump pulse Δ*τ*_pump_=32±0.5 fs), we see a further and strong increase of spectral weight in the Ti 3*d* band up to 200 fs (red data in Fig. 1g). Because *k*-dependent spectral weight effects would be expected to lead to a decrease of the photoemission intensity^14^,^23^ (Supplementary Note 1), the observed increased spectral weight can rather be linked to an increase in the number of free carriers in the observed Ti 3*d* band, as has also been observed recently in a time-resolved infrared pump—THz probe measurement by Porer *et al*.^16^.

Figure 1g further displays the decrease in spectral weight of the backfolded Se 4*p* band (blue data, plotted inverted) as a function of time, which is indicative for the CDW quenching and is also associated with the closing of the gap^13^,^15^,^23^, even in the case of non-equilibrium conditions^24^. Note that these spectral weight data are plotted as an increase (that is, mirrored around the *x* axis) to enable a better comparison with the increase in spectral weight of the Ti 3*d* band. The initial response times of the CDW quenching as a function of laser fluence, which are evaluated from the Se 4*p* band suppression, are plotted in Fig. 1h (blue data points). We fit our data with *τ*=*c* × *F*^*x*^, where *c*=44.6±2.1 fs cm^2^ mJ^−1^ is a constant, *x*=0.29±0.04, and *F* represents the absorbed pump fluence that can be assumed to be proportional to the number of generated free carriers. We and others have shown previously that the number of optically generated free carriers is a critical parameter for the ultrafast quenching of the CDW phase, assuming an interpretation based on screening^15^,^16^,^25^. However, the qualitative relation between the number of optically generated free carriers and the CDW quenching does not elucidate the role of the transient population and dynamics of the excited states. In particular, we have already seen from Fig. 1 that the number of free carriers does not remain constant after the 32 fs laser pulse excitation, but rather increases for timescales up to about 200 fs. We therefore focus in the rest of this article on an analysis of the hot electron dynamics and free carrier generation: how these dynamics depend on the gap size, and how they impact the CDW quenching in the case of 1*T*-TiSe_2_.

### Momentum-resolved dynamics

**Figure 2:**
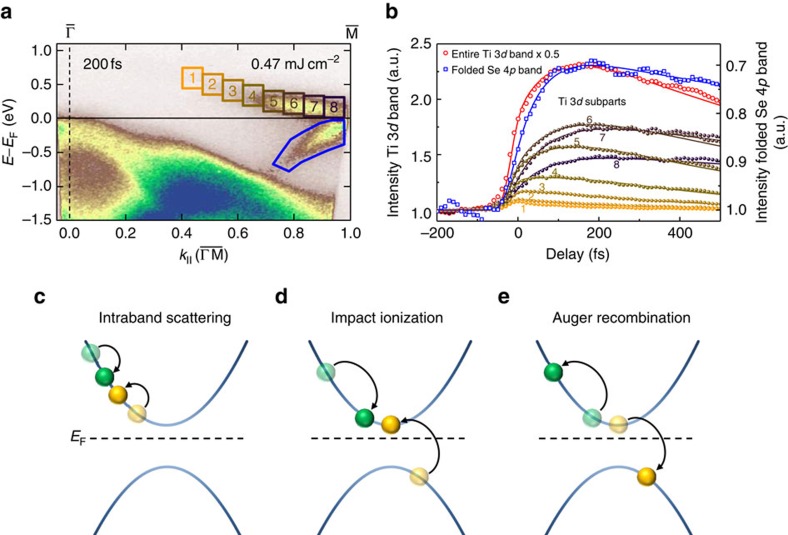
Hot-electron dynamics in the Ti 3*d* band. (**a**) ARPES map of TiSe_2_ at *t*=200 fs after excitation with an absorbed fluence of 0.47 mJ cm^−2^. (**b**) Spectral weight in the Ti 3*d* band as a function of time in comparison to the quenching of CDW. (**c**–**e**) Possible electron–electron scattering processes: intraband scattering (**c**), and interband impact ionization scattering (**d**) as well as the reverse process, Auger recombination (**e**). Note that the phase space for impact ionization scattering is increased for smaller gap sizes, where more scattering processes with smaller energy transfer become possible.

Figure 2 displays the transient behaviour of the photo-excited hot electrons for different energies and momenta in the Ti 3*d* band, for time delays between the pump and probe pulses of up to *t*=500 fs. The transitions involved in the optical excitation process are explained in detail in refs 15, 21, 22. The general picture of the dynamics is the following. After optical excitation from the Se 4*p*_x,y_ bands to the Ti 3*d* band, the initial non-equilibrium (non-Fermi-Dirac) distribution starts to thermalize due to electron–electron scattering, and cools via electron–phonon scattering, and recombination. These processes occur on different timescales, and act differently on the electron distribution. In this article, we will now concentrate on the very fast timescales, where electron–electron scattering processes are mostly dominant.

For electron–electron scattering, several processes are possible, as shown in Fig. 2c–e. Hot electrons can loose energy via Coulomb scattering with other electrons in the same band (intraband scattering within the Ti 3*d* band, Fig. 2c), or with electrons in a different band located below the Fermi level (interband scattering between the Ti 3*d* and the Se 4*p* bands, Fig. 2d). In both cases, the scattered electron gains energy and momentum from the hot electron and is excited into an unoccupied state. If this second electron originates from a Se 4*p* band below the Fermi-level and is excited across the gap into the Ti 3*d* band, such a scattering event creates an additional free carrier in the material and is called impact ionization^26^, which has just recently been shown to be a very important process in graphene^27^,^28^,^29^. Conversely, hot electrons can also gain energy via the reversed process, that is, Auger recombination (Fig. 2e). While impact ionization leads to an increase of the number of free carriers and a thermalization of the electrons, Auger recombination, in contrast, decreases the number of free carriers and counteracts the thermalization process. As seen in Fig. 2b, we find a strong increase in the number of carriers up to 200 fs (red data and line) that we interpret as a signature of impact ionization, as we explain in more detail below.

In a qualitative picture, electrons scatter down the Ti 3*d* band via electron–electron scattering (compare spectral weight in integration areas 1–8 in Fig. 2a,b). Since in an impact ionization event, the energy of the gap must be overcome by the second electron, the size of the gap determines the minimum energy transfer needed in each scattering event, as shown in Fig. 2d. Moreover, these interband electron–electron scattering events are very efficient as long as the electron distribution exhibits a non-equilibrium distribution with many unoccupied states at the bottom of the conduction band, which are available for the in-scattering process. As soon as the electron distribution is relaxed to a Fermi-Dirac distribution, however, these states become occupied, and electron–electron scattering processes into these states are suppressed.

At this point, we need to define our terms for the different stages of electronic dynamics. In this article, we will refer to the initial optically induced non-equilibrium non-Fermi-Dirac electron distribution as the non-equilibrium distribution. As will be shown below, this non-equilibrium distribution takes ∼200 fs to thermalize into a hot, Fermi-Dirac electron distribution via electron–electron scattering. We will call this a quasi-equilibrium with a characteristic temperature and a quasi Fermi-level (Fig. 1e), in accordance with semiconductor physics terminology^30^. Moreover, we call the non-equilibrium (non-Fermi-Dirac) to quasi-equilibrium (Fermi-Dirac) relaxation of the electron gas a thermalization, while we denote the equilibration of electron and lattice temperatures by cooling.

Focusing on the non-equilibrium to quasi-equilibrium thermalization, the increase in spectral weight of the Ti 3*d* band well after optical excitation indicates that impact ionization plays a dominant role. In contrast to impact ionization, other scattering processes, such as intraband, Auger, and electron–phonon scattering, while definitely also contributing to the dynamics, cannot cause an increase in spectral weight of the Ti 3*d* electron state, in contrast to our observation. We also note that impact ionization processes strongly depend on the size of the energy gap, because the electrons in the Ti 3*d* band must lose at least the band-gap energy in this scattering process. However, the energetic position of the (quasi-particle) bands involved may change after impulsive optical excitation, leading to a renormalization of the (quasi-particle) energy gap. For TiSe_2_, in particular, although not fully quenched, the CDW energy gap rapidly decreases after optical excitation^16^. Therefore, the available phase space for electron–electron interband scattering critically depends not only on the band structure of the unexcited system, but also on the dynamics of the characteristic quasi-particle energy gaps in the material.

### Numerical results

**Figure 3:**
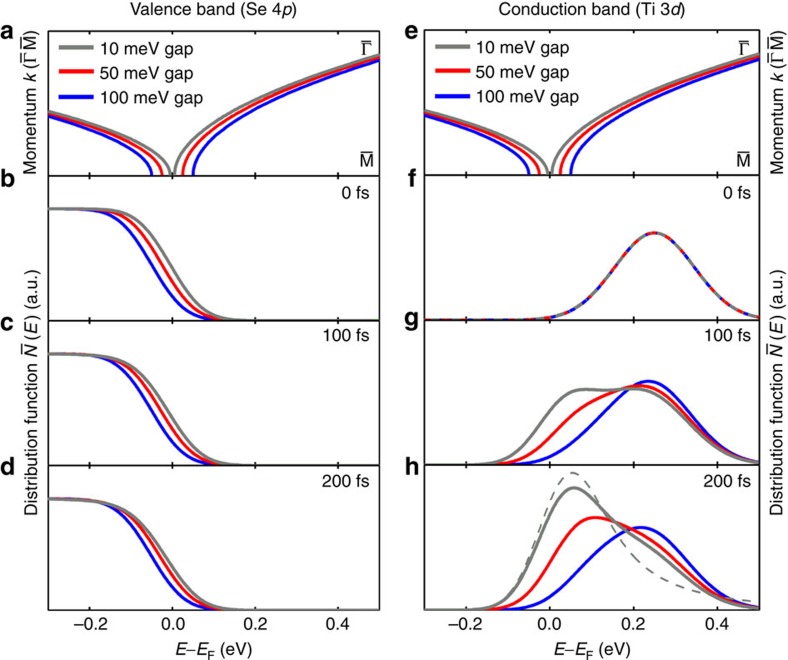
Computed dependence of carrier dynamics on the size of the gap. (**a**,**e**) Band lineup for small gap (10 meV, grey lines), intermediate gap (50 meV, red lines), and large gap (100 meV, blue lines). Note that the holes that have been created in the optical excitation process far below the Fermi-level (between *E*–*E*_F_≈−0.9 and −1.5 eV) are not included. (**b**–**d**) Computed dynamical electron distributions in the valence band and (**f**–**h**) computed electron distributions in the conduction band (right, six times amplified in comparison to **b**–**d**), broadened with the experimental energy resolution: initial distributions (**b**,**f**) and results after 100 fs (**c**,**g**) and 200 fs (**d**,**h**). At a 200 fs delay, the dynamical electron distribution in the conduction band in the case of a small gap (solid grey line) is already very close to a quasi-equlibrium distribution (dashed grey line), as the low energy states in the conduction band are filled by additional carriers created via impact ionization. In contrast, the dynamical electron distribution for the large gap case (blue lines) prevents efficient relaxation via impact ionization between the conduction and valence bands toward a quasi-equilibrium distribution on femtosecond timescales.

To assess the importance of the band-gap renormalization on the non-equilibrium hot carrier dynamics, we numerically analyse electron dynamics in a two-band model with different gap sizes. The momentum-resolved carrier distributions are computed for an effective two-band system with two-dimensional *k*-space including Boltzmann scattering integrals for the electron-electron interaction as well as density-dependent screening (in a static approximation, for details see Supplementary Fig. 2–4 and Supplementary Methods). Figure 3a–d shows the calculated evolution of the carrier density in the valence band (which represents the backfolded Se 4*p* bands) and Fig. 3e–h the calculated evolution of the conduction band (which represents the Ti 3*d* band) as a function of time for three different gap sizes (Δ*E*_gap_=10, 50 and 100 meV). The computed distribution functions have been broadened by a Gaussian with the experimental energy resolution (150 meV). Due to the simplicity of the model, we cannot compute a realistic total experimental photoexcitation process including matrix elements. Instead, we therefore take the spectral dependence of the carrier distributions, broaden them with the experimental energy resolution, and take this as an approximate measure of the experimental signal. Let us focus first on the excited electrons in the conduction band (Fig. 3f–h). For each gap size, the initial distribution is the same distribution peaked at ∼0.25 eV (Fig. 3f). After 200 fs (Fig. 3h) and for a gap size of 10 meV (grey curve) the initial distribution has completely changed and the maximum has shifted towards the electronic states at the band bottom; in fact, the quasi-equilibrium distribution is already nearly established, however, with a much larger area under the computed *N*(*E*) curve, that is, an increased number of carriers. In contrast, the initial non-equilibrium electron distribution (Fig. 3f) persists in the case of the large 100 meV gap (Fig. 3h, blue curve), even at times much longer than 200 fs (not shown).

Note that the peaks of the initial distributions in Fig. 3f coincide for the three band structures shown in Fig. 3e. Figure 3g,h thus highlight the different subsequent relaxation dynamics for the different band gaps. For instance, for a static gap of 50 meV the computed carrier distributions show a peak shift and a carrier multiplication similar to the experimental results. However, the energetic position of the peaks cannot be mapped precisely to the experiment, as we do not take into account modifications of the band dispersion near the band edges due to hybridization of Se 4*p* and Ti 3*d* states^23^.

Remarkably, for the valence electron dynamics, Fig. 3b–d, there is no strong dependence of the carrier dynamics on the gap, because at the top of the valence band, the initial distribution 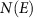 of the electrons in the valence band is Fermi-Dirac like. This is because the optical excitation originates from the parallel aligned Se 4*p*_x,y_ bands located at 1.6 eV below the Ti 3*d* band in the second half of the Brillouin zone in 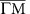 direction^15^,^21^,^22^ (see purple arrows in Fig. 1b, 0 fs snapshot), and therefore well below the characteristic energies of the backfolded Se 4*p* bands. In addition to the weak dependence of the carrier dynamics in the valence band, we also see that the carrier dynamics affect the backfolded Se 4*p* valence band only very close to the top of the band. We note that this change is not captured in the experimental data, where we analyse the backfolding suppression away from the 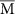 point (see blue area and the location of the 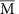 point, which is outside of the measured photoemission momentum area in Fig. 1d, and Supplementary Note 2). We can therefore summarize here that the evolution of the initial non-equilibrium carrier distribution should critically depend on the characteristic gap size of the material. For electron thermalization processes from non-equilibrium to quasi-equilibrium, our calculation implies that impact ionization processes are essential, and that the efficiency and speed of the thermalization process depend on the gap size.

## Discussion

In the case of 1*T*-TiSe_2_, we now argue that this gap-size dependent non-equilibrium behaviour explains—on a microscopic level—the extremely fast response of this CDW material to an optical excitation. First, we note that the quenching of the CDW in 1*T*-TiSe_2_ evolves on similar timescales as the carrier multiplication process, that is, the CDW gap size is dynamically reduced^15^,^16^. From our calculations, we infer that a reduced gap size will dynamically amplify the process of carrier multiplication. An increased density of the conduction electrons, on the other hand, will induce screening and thus quench the excitonic CDW insulator phase in 1*T*-TiSe_2_, see refs 15, 16. Quenching of the CDW phase is equivalent to further closing of the gap, which will make carrier multiplication even more effective. Thus, we have a mutual amplification of carrier multiplication, screening and quenching of the CDW (closing of the CDW gap). In terms of timescales, however, carrier multiplication processes can only evolve until the quasi-equilibrium is reached. In quasi-equilibrium, much less phase space for interband electron—electron scattering is available, and thus the process of carrier multiplication stops. There is no additional build-up of screening, so that the quenching of the CDW phase in TiSe_2_ stops as well. As a consequence, we expect that the non-equilibrium to quasi-equilibrium thermalization timescale dictates the speed of the CDW quenching. We will now validate this conjecture by analysing the experimental data.

**Figure 4:**
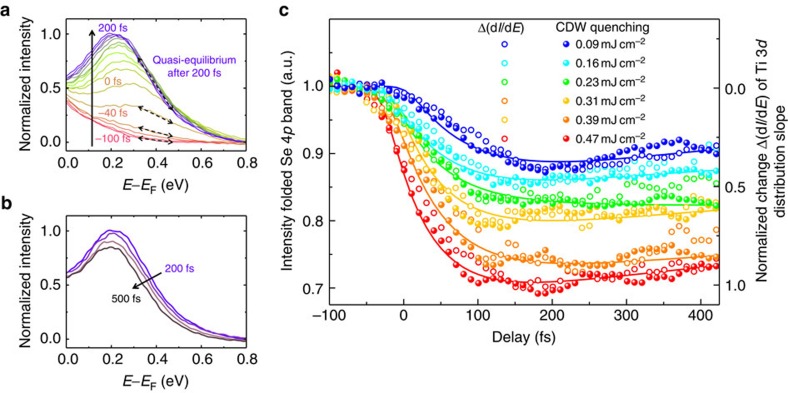
Non-equilibrium electron dynamics drive the ultrafast quenching of the CDW in TiSe_2_. (**a**) Measured energy distribution curves (EDC) of the Ti 3*d* band as a function of time in 20 fs steps from −100 to 200 fs for an absorbed fluence of 0.47 mJ cm^−2^ (red to blue lines, *k*_||_-integration from 0.23 to 1.05 Å^−1^). First, the optically induced non-equilibrium electron distribution relaxes via electron–electron scattering processes to a hot Fermi-Dirac quasi-equilibrium in the Ti 3*d* band, which is reached after ≈200 fs. This non-equilibrium to quasi-equilibrium thermalization process is illustrated in the data by a fast change of the slope Δ(d*I*/d*E*) of the distribution at *E*−*E*_F_=0.4 eV (black dashed double arrows). (**b**) Subsequently, the system cools via electron–phonon scattering and recombination, which is visible in the 200–500 fs data in 100 fs steps by a subsequent, slower change of the slope (that is, temperature), and an energetic lowering of the elevated quasi Fermi-level. Note that the EDC peaks are at a higher energy above *E*_F_ in comparison to the theoretical result in Fig. 3, because the computed carrier dynamics have been plotted for the fixed band structures in Fig. 3e to highlight the different relaxation dynamics for the different gap sizes. (**c**) Comparison of hot-electron thermalization (analysed via extracting the change of the slope of the electron distribution Δ(d*I*/d*E*), open circles) and suppression of backfolding intensity from the backfolded Se 4*p* bands (filled circles), which are indicative of the quenching of the CDW, as a function of time for different pump fluencies. The lines are exponential decay fits to the CDW quenching (the respective time constants are shown in Fig. 1h).

In our data, the thermalization process can be monitored by following the change of the slope of the integrated electron distribution Δ(d*I*/d*E*) of the hot electrons in the Ti 3*d* band during the first 200 fs (Supplementary Note 3). Figure 4a displays energy distribution curves (EDCs) of the momentum-integrated Ti 3*d* band as a function of time between -100 and 200 fs. Once again, one sees that the electrons are excited by the pump pulse, if one compares the EDC at negative delay with the EDCs around time zero. As long as electron–electron scattering processes are very efficient—which is the case during the thermalization process—the slope of the distribution d*I*/d*E* will change rapidly. Note that one cannot directly convert the change of the slope to a temperature scale, since the electron distribution is in a non-equilibrium state during the thermalization process. As soon as the electron system is thermalized to a quasi-equilibrium however, much less phase space for electron–electron scattering is available, and the rapid change of the slope of the distribution Δ(d*I*/d*E*) should stop, as described theoretically above. Indeed, we can see directly in our data that within the first 200 fs, a very rapid thermalization via carrier multiplication occurs (fast change of slope, increase of spectral weight in the Ti 3*d* band). The rapid changes in the slope of the distribution due to electron–electron scattering terminate around *t* ≈ 200 fs, when a Fermi-Dirac quasi-equilibrium has been reached. Also, we see that this state is characterized by an electron distribution with an elevated quasi Fermi-level in comparison to a fully relaxed system^30^ (purple dashed line in the 200 fs photoemission map of Fig. 1e). The extracted temperature of the electron gas at this time is still very high at about 750–1,000 K, depending on fluence. This hot electron gas then cools (*t*>200 fs) via electron–phonon scattering and recombination, which is observed by slow further gradual steepening of the slope of the Fermi distribution and a lowering of the quasi Fermi-level shown in Fig. 4b.

To elucidate whether the non-equilibrium to quasi-equilibrium thermalization time dictates the speed of the CDW quenching in TiSe_2_, as we proposed above, we now plot the change of the slope in the EDCs Δ(d*I*/d*E*) (open circles) together with the CDW quenching (filled circles) in Fig. 4c. Note that the CDW quenching is analysed from the spectral weight suppression of the folded Se 4*p* band away from the Se 4*p* band maximum, so that holes created via impact ionization do not contribute to this signal (Supplementary Note 2). Indeed, the characteristic timescales for non-equilibrium to quasi-equilibrium thermalization and CDW quenching coincide for all fluences. Moreover, due to the mutual amplification of carrier multiplication and CDW quenching, an extremely strong fluence dependence can be expected. At high fluence, not only the total number of optically excited carriers in the Ti 3*d* band is larger than at low fluence, but also a larger number of carriers are excited into the Ti 3*d* band at earlier times within the temporal width of the pump pulse (Δ*τ*_pump=_32±0.5 fs). Therefore, it can be expected that not only the initial speed-up of carrier multiplication and CDW quenching is stronger in the high-fluence regime, but also that the process itself starts at earlier times. Indeed, all of these temporal signatures are found in our data (Fig. 4c). The higher the applied pump fluence is, the earlier the dynamics starts and the stronger the initial speed-up develops. These very fast initial processes are then subsequently counteracted by a reduction of the electron–electron scattering phase space, until a thermalized electron gas is reached.

The microscopic picture developed here to describe the dramatic response of 1*T*-TiSe_2_ to pulsed optical excitation is consistent with the idea of charge-transfer excitonic correlations^31^, which we used previously to interpret trARPES data^15^, and which has been shown to be also consistent with recent THz measurements^16^ on TiSe_2_. In particular, the THz measurements by Porer *et al*.^16^ already suggested that the pump-generated primary electron–hole pairs are multiplied via cascaded electron–electron scattering processes, and we indeed have observed these processes here in real time and in a direct manner using photoemission spectroscopy. In addition to the interpretation of Porer *et al*.^16^, who postulated that increased screening induces a break-up of bound pairs into free carriers, our direct time-resolved photoemission data elucidates that free carriers are dominantly generated at elevated energies in the Ti 3*d* band, caused by impact ionization processes. The observation of additional carriers due to impact ionization, moreover, is also consistent with recent theoretical work by Golez *et al*.^24^, where, however, impact ionization is driven via the break-up of excitons and the mechanism for melting of the gap is proposed to be caused by a transfer of kinetic energy to the condensate rather than due to screening. In addition to the role of impact ionization, the theoretical model by Golez *et al*.^24^ nonetheless also predicts a direct connection between thermalization and cooling of the carriers with the CDW melting, which is in agreement with our observation, as shown in Fig. 4c. In consequence, impact ionization is connected to the melting of the gap, partially through an enhanced screening due to the increased number of carriers, as seen here, and possibly also through a direct transfer of kinetic energy to the exciton condensate via inelastic scattering, as suggested by Golez *et al*.^24^.

Before concluding the paper, we would like to assess the role of the lattice in our interpretation, which we have omitted so far. In particular, previous works show that it is likely that the CDW gap in TiSe_2_ is jointly formed by excitonic correlations and Jahn-Teller effects^16^,^31^, so that dynamics of the gap can be expected to evolve both on electronic and phononic timescales. Indeed, given recent measurements of the A_1g_ amplitude mode in TiSe_2_ after impulsive optical excitation^3^,^16^, we see that a quarter of a coherent oscillation can already be completed within 250 fs. It is therefore likely that a part of the dynamics that we observe is not only driven by electronic, but also by phononic contributions. However, two observations indicate that electronic processes are the main driving force for CDW quenching within the first 200 fs. First, we have seen previously that a quenching of the CDW can be as fast as 20 fs^15^ (see also Fig. 1h), which must clearly be assigned to a purely electronic process and is fully consistent with the electron-based interpretation developed here. Second, our electron-based interpretation explains very intuitively the dramatic fluence-dependent speed-up of the CDW quenching, while the fluence-dependence of the frequency of the amplitude mode^3^,^16^ would rather suggest that the dynamics slow down.

In summary, we have investigated ultrafast non-equilibrium electron dynamics in a correlated material and how they depend on the characteristic energy gap. Our first finding is that carrier multiplication via impact ionization can play a dominant role in the initial non-equilibrium dynamics, in a similar fashion to what has been found in semiconductors and more recently in graphene. These inelastic electron–electron scattering processes are responsible for the transition from the photo-induced non-equilibrium electron distribution to a quasi-equilibrium hot Fermi gas. Most importantly, the efficiency of carrier multiplication and thermalization critically depends on the characteristic gap size, which may dynamically change after photoexcitation. In the reference system 1*T*-TiSe_2_, our momentum-resolved findings can explain on a microscopic level the dramatic time-dependent response of this CDW material to an optical excitation. Our observations emphasize the importance of non-equilibrium carrier dynamics in ultrafast photo-induced phase transitions, and show how, in general, non-thermal dynamical pathways can play a dominant role in the ultrafast response of correlated electron materials.

### Data availability

All relevant data are available from the authors on request.

## Additional information

**How to cite this article:** Mathias, S. *et al*. Self-amplified photo-induced gap quenching in a correlated electron material. *Nat. Commun.* 7:12902 doi: 10.1038/ncomms12902 (2016).

## Supplementary Material

Supplementary Information Supplementary Figures 1 – 6, Supplementary Notes 1 – 3, Supplementary Methods and Supplementary References
