# Supplementary material for: Self-amplified photo-induced gap quenching in a correlated electron material
Source: Nat Commun. 2016 Oct 4;7:12902. doi: 10.1038/ncomms12902 (PMC5059442; doi:10.1038/ncomms12902)
Supplement: Supplementary Information — Supplementary Figures 1 – 6, Supplementary Notes 1 – 3, Supplementary Methods and Supplementary References [file ncomms12902-s1.pdf]

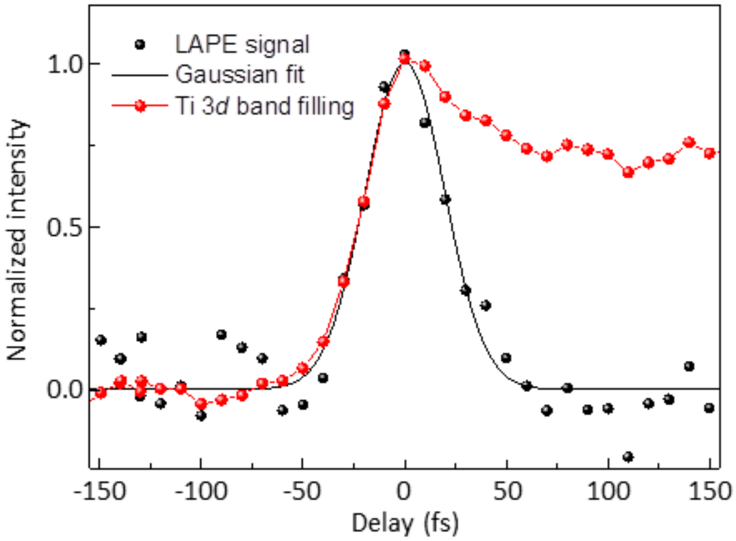

Supplementary Figure 1: Determination of time zero and time resolution using the laser-assisted photoelectric effect (LAPE). The black data points represent the temporal evolution of the LAPE sideband at 1.6 eV above the folded Se 4*p* band at the $\overline{\mathrm{M}}$ point for a fluence of 0.97 mJ cm^{-2}. The red data points show the transient filling of the energetically uppermost part of the Ti 3*d* band. Both features reach their intensity maximum at the same time delay, which corresponds to time zero of the experiment. Therefore, at lower pump fluencies, where no LAPE sidebands are created, we used the maximum filling of the high-energy part of the Ti 3*d* band for the determination of time zero. We note that the fluence used here was high sufficiently high to extract the LAPE signal and to determine time zero and the time resolution of the experiment, however, accompanying severe space charge effects did prevent a detailed analysis of the data as has been presented in the main manuscript for lower fluencies. We fit the measured LAPE signal with a Gaussian resulting in a full width at half maximum (FWHM) of $\Delta\tau_{\mathrm{LAPE}} = 46 \pm 8$ fs. The near-infrared pump pulse duration was measured with frequency-resolved optical gating (FROG) and has a FWHM of $\Delta\tau_{\mathrm{pump}} = 32\ \pm 0.5$ fs. From those values we calculate the HHG probe pulse duration to be $\Delta\tau_{\mathrm{HHG}} = 33\ \pm 12$ fs. The error bars of the pump pulse and the LAPE duration arise from the Gaussian fit to the experimental data, while the error bar of the HHG pulse duration is a consequence of the error propagation from the two other values.

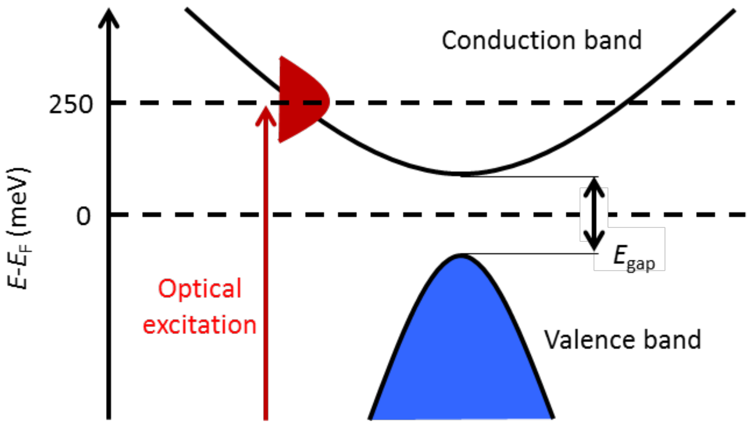

Supplementary Figure 2: Schematic diagram of the conduction and valence band lineup as well as the initial excitation conditions for our theoretical model.

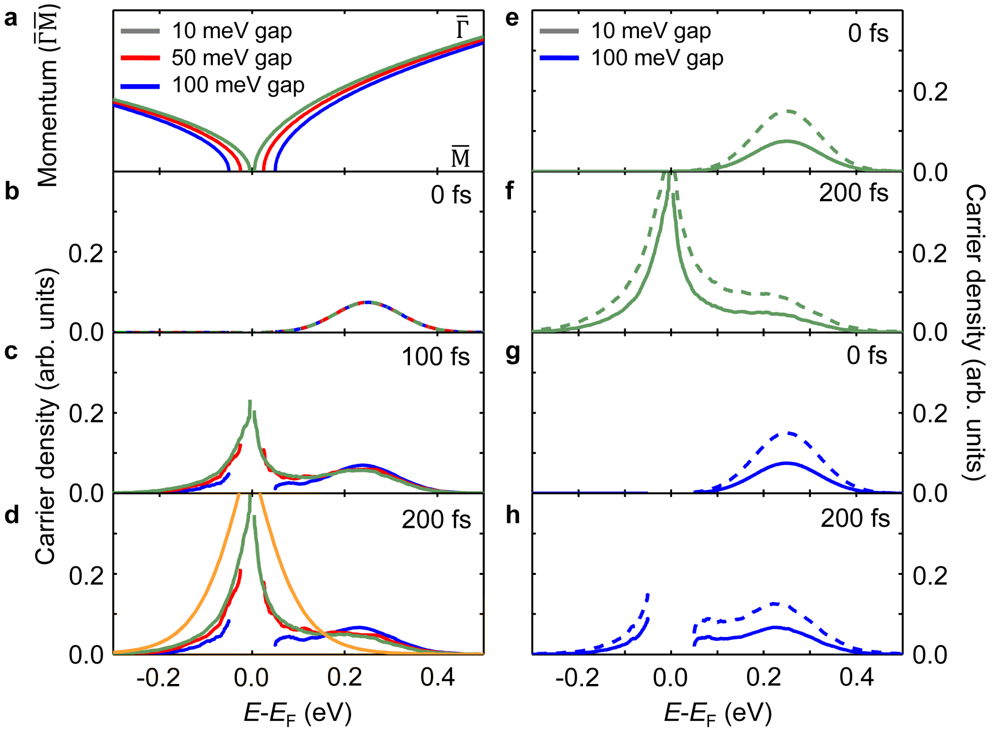

Supplementary Figure 3: Band structure and carrier distributions for theoretical model. (a) Band structure $E(k)$ for a band gap of 10, 50, and 100 meV. **(b–d)** Computed carrier distributions for these gap sizes at 0 fs (b), 100 fs (c), and 200 fs (d). The orange curve in (d) indicates the quasi-equilibrium distribution. **(e-h)** Computed carrier distributions at 0 fs (a) and 200 fs for two excitation densities of N_1^{D} (solid lines, same as in b-d) and $N_2^{\mathrm{D}} = 2N_1^{\mathrm{D}}$ (dashed lines). (e,f) gap size of 10 meV ; (g,h)) gap size of 100 meV. Note that the quasi-equilibrium distribution here is computed including impact ionization, whereas for the quasi-equilibrium curve shown in Fig. 3 in the main text, impact ionization was neglected after 200 fs.

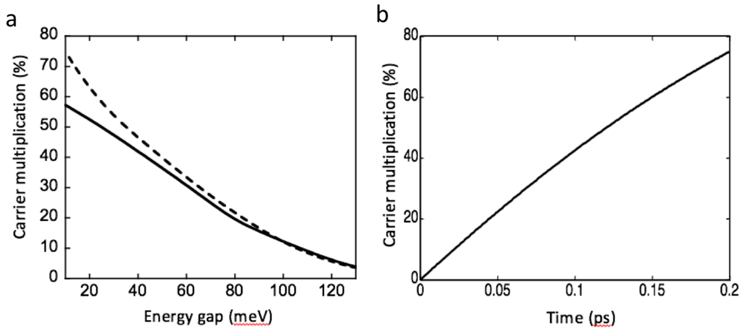

Supplementary Figure 4: Carrier multiplication. (a) Carrier multiplication vs. gap size after 200 fs for scenario for excited densities N_2^{D} (solid line) and N_1^{D} (dashed line). **(b)** Carrier multiplication vs time is plotted for a gap size of 10 meV for the small density.

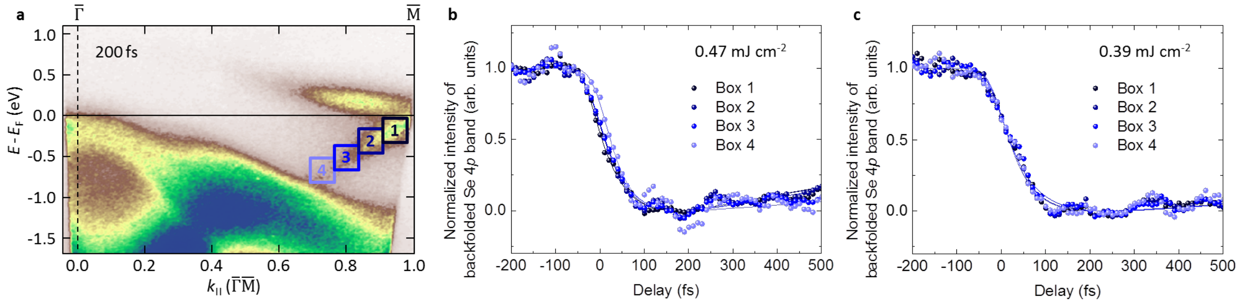

Supplementary Figure 5. Momentum-dependent quenching of the intensity in the backfolded Se 4*p* band. (a) 200 fs snapshot that indicates the integration areas 1-4. **(b,c)** Normalized photoemission intensities of the backfolded Se 4*p* band in areas 1-4 for (b) a fluence of of 0.47 mJ cm^{-2}, and (c) a fluence of 0.39 mJ cm^{-2}. All transients 1-4 are identical within error. We therefore conclude that holes created via impact ionization, which would show a $k_{||}$-dependence, do not contribute to the loss of spectral weight away from the $\overline{\mathrm{M}}$-point.

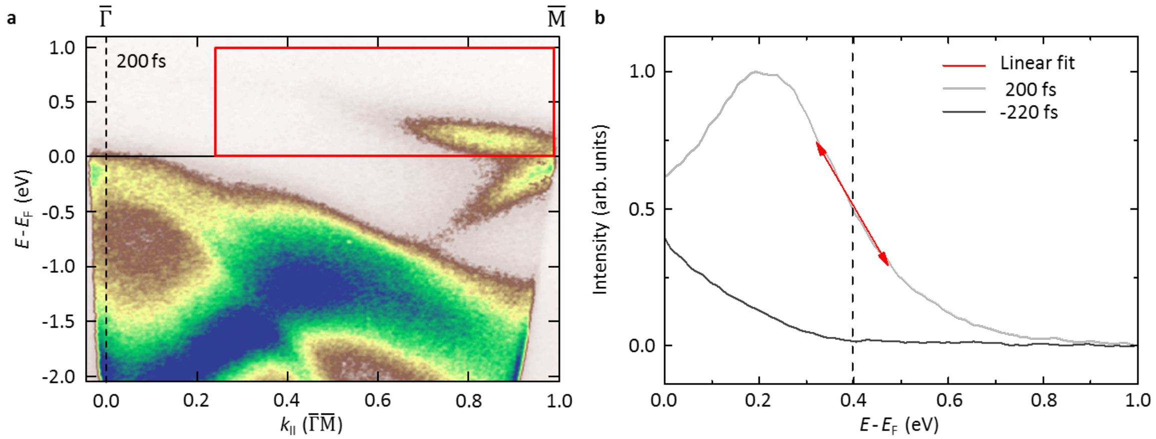

Supplementary Figure 6: Slope extraction of the Ti 3*d* band excitation. (a) illustrates the integration area in the ARPES maps for the EDCs of the excited Ti 3*d* band. **(b)** shows EDCs for the not yet excited band at -220 fs (dark gray line) and the band with the highest electron filling at 200 fs (light gray line).

Supplementary Note 1: k-dependent spectral weight effects in the unoccupied Ti 3*d* band. In the main manuscript, we focus on the reason for the increase of spectral weight in the Ti 3*d* band at times beyond the excitation pulse, and conclude that impact ionization is the most likely responsible process. We note that usually an increase in the spectral function in a correlated material can be due to many different band renormalization processes, see for instance ref. 1 for the case of 1*T*-$TiSe_2$. However, the relevant quantity that changes the photoemission intensity is not the total number of states, but the total number of electrons occupying these states. With respect to the occupation of states with electrons, however, it is imperative to distinguish band renormalization processes in the occupied and in the unoccupied regime of the band structure, below and above the Fermi-level, respectively.

For states below the Fermi-level, a band renormalization that changes transiently the density of states in a specific energy interval will always go along with a redistribution of electron density. This redistribution will in consequence also affect the measured photoemission intensity.

Far above the Fermi-level, similar effects (due to band renormalization) are to be expected. But in addition, one has to consider the specific electronic states that are occupied either through the photo-excitation process or the following scattering process. Since photo-excitation happens between the Se $4p_{x,y}$ bands and the Ti 3*d* band in $\overline{\Gamma}$-$\overline{\mathrm{M}}$ direction (see purple arrows in Fig. 1b), there are no additional electrons anywhere else in the energy-momentum space right after the excitation. Therefore, a possible transient band renormalization can only result in a decrease of electron population in the probed $E(k_{\parallel})$-area, but not in an increase. This leaves scattering processes as the only mechanism capable of creating additional hot electrons far above the Fermi level, and these scattering processes are discussed in the main manuscript.

Supplementary Note 2: Spectral weight in the backfolded Se 4*p* band. The spectral weight in the backfolded Se 4*p* band around the $\overline{\mathrm{M}}$ point is analyzed in our manuscript to follow the transient and ultrafast quenching of the CDW phase in 1*T*-$TiSe_2$. At the same time, we argue that impact ionization is an efficient electron-electron scattering process in this material, which transfers spectral weight from the occupied Se 4*p* band to the unoccupied Ti 3*d* band. This raises the question in how far the created holes in the Se 4*p* band show up in our data, and how the analyzed quenching of the CDW is altered due to this process. We will show now that our signal is free of additional loss due to impact ionization for the $E(k_{\parallel})$ area that we used for the analysis of the suppression of the backfolded Se 4*p* band.

In the process of impact ionization the electron in the Ti 3*d* band must have excess energies higher than the energy gap plus the binding energy of the respective scattering partner in the Se 4*p* band (binding energy with respect to the top of the Se 4*p* band). Therefore, the creation of holes due to impact ionization in the Se 4*p* band is strongly **k**-dependent, and most efficient at the top of the Se 4*p* band. These considerations are confirmed by our theoretical calculations (Figure 3), where the largest hole density is established at the very top of the Se 4*p* band.

In our experiment, we analyzed the suppression of the backfolded Se 4*p* band not at the top of the Se 4*p* band, but at $E(k_{\parallel})$ values away from the $\overline{\mathrm{M}}$ point. Since, as explained above, the loss of spectral weight due to impact ionization can only be seen at the top of the band (*i.e.*, at the $\overline{\mathrm{M}}$ point), our analysis procedure should only reflect a loss of spectral weight due to CDW quenching. Experimentally, it is possible to confirm this consideration via a **k**-dependent analysis. Supplementary Figure 5 shows four $k_{\parallel}$-

resolved transients of the Se 4*p* band as a function of time that represent the area that we used for the analysis of the suppression of the backfolded Se 4*p* band intensity. For the arguments given above, it is a reasonable assumption that far away from the $\overline{\mathrm{M}}$ point hole creation via impact ionization is not an efficient process (*i.e.*, at high binding energies, see dataset 4), and the suppression of spectral weight must solely be attributed to CDW quenching. If the creation of holes via impact ionization could be seen in our data, the transients 3-1 should show a $k_{||}$-dependence, which is, as shown in Supplementary Figure 5, clearly not the case. Therefore, it is evidenced that the loss of spectral weight in the $E(k_{||})$ area that we used for analysis is a reliable measure for the CDW quenching.

We note that due to insufficient energy resolution it was not possible to experimentally analyze the loss of spectral weight at the top of the backfolded Se 4*p* band (here, Se 4*p* and Ti 3*d* bands overlap within our energy resolution), where both CDW suppression and impact ionization should contribute to the signal.

Finally we want to point out that if a substantial amount of holes due to photo-excitation and/or impact ionization would be present in the Se 4*p* band, Auger scattering instead of impact ionization (which is inverse Auger scattering) would be the dominant electron-electron scattering process (see also Ref. 2). In the case of Auger scattering, however, one would expect an increase of the average kinetic energy in the Ti 3*d* band, and a decrease of the number of carriers, which is the opposite behavior in comparison to our observations. Furthermore, as a function of fluence, dominant Auger scattering would induce a slow-down of the non-equilibrium to quasi-equilibrium thermalization time, which is also in contrast to our measurements, where we see a drastic speed-up.

Supplementary Note 3: Extraction of the non-equilibrium to quasi-equilibrium thermalization time. For the determination of the non-equilibrium to quasi-equilibrium thermalization time, we analyze EDCs that are $k_{||}$-integrated from 0.23 Å^{-1} to 1.05 Å^{-1} (see Supplementary Figure 6a, red box). By plotting those EDCs at different time delays in 20 fs steps from -100 fs to 200 fs and in 100 fs steps from 200 fs to 500 fs, we obtain Fig. 4a and b from the main text. Supplementary Figure 6b shows two EDCs at -220 fs (before excitation) and at 200 fs, where the maximum filling of the Ti 3*d* band occurs (fluence of 0.47 mJ cm^{-2}). Because the electronic system is in a non-equilibrium state, it is not reasonable to fit a Fermi-Dirac function to our data. Therefore, we monitor the high-energy slope of the distribution $\mathrm{d}I/\mathrm{d}E$ as a function of time, which is used as a measure of the thermalization process of the carriers in the Ti 3*d* band into a quasi-equilibrium. After data normalization, we use the half intensity in the 200 fs EDC as the center point for the slope extraction (black dashed line). For sufficient statistics, 10 data points in both energy directions were added to fit a linear function (red arrow in Supplementary Figure 6b). By using the same energy region for all other time steps within one fluence and extracting the slope of the linear fit function, we received the change of the slope $\Delta(\mathrm{d}I/\mathrm{d}E)$ over time as shown in Fig. 4c in the main text. After the electronic distribution is thermalized, it is reasonable to carry out a Fermi-Dirac fit, and we extract electron temperatures between 989 ± 16 K for the highest fluence (0.47 mJ cm^{-2}) and 744 ± 15 K for the lowest fluence (0.09 mJ cm^{-2}).

Supplementary Methods: The theoretical model

Here, details are presented on the calculation of the carrier dynamics in a two-band system with a gap of variable size. The computed thermalization dynamics and the efficiency of carrier multiplication show a pronounced dependence on the gap size. This result supports the conclusion presented in the main text that observed ultrafast carrier and impact ionization dynamics in 1*T*-$TiSe_2$ are due to a transient renormalization of the band gap.

In our model, we calculate the time-dependent single-particle distribution functions for electrons in a two-band model with a fixed gap between a conduction and valence band and an effectively two-dimensional *k*-space. We take into account dynamical electron-electron Coulomb scattering at the level of Boltzmann scattering integrals. This model is designed to describe the ultrashort-time dynamics far from equilibrium, as it is driven by optical excitation with an ultrashort pulse. The Coulomb scattering mechanism is responsible for carrier multiplication processes and sets the time scale at which a hot quasi-equilibrium electron distribution is reached. We do not include electron-phonon scattering and recombination processes, which would be necessary to describe the cooling of the hot electron gas to room temperature and the establishment of an equilibrium between all the bands involved in the initial optical excitation process. These latter cooling processes take place on a longer timescale than that considered in our calculations.

I. Model Description:

We consider two spin-degenerate conduction and valence bands, which are labeled by a band index $b = \{c, v\}$, with isotropic parabolic dispersions

$$\varepsilon_{\mathrm{b}}(k) = \frac{\hbar^2}{2m_{\mathrm{b}}} k^2 \qquad \text{Supplementary Equation 1}$$

and effective masses. Note that the wave vector **k** is two-dimensional and that we do not take spin-mixing due to the spin-orbit interaction into account.

The band lineup at the $\overline{\mathrm{M}}$ point, which is equivalent to the $\overline{\Gamma}$ point in the CDW phase, is actually more complicated than this. As described by Monney *et al.*[3], there are one valence band (mainly of Se 4*p* character) and three elliptically shaped conduction bands (mainly of Ti 3*d* character) directly at the $\overline{\mathrm{M}}$ point. In our treatment we simplify this band structure by choosing effective masses for one conduction and one valence band.

We calculate the time evolution of $n_{\mathrm{b}}(k)$, which is the distribution of carriers in band b at momentum $\mathbf{k}$ by solving the dynamical equation with Boltzmann scattering terms in the following form

$$\frac{\partial}{\partial t} n_{\mathrm{b}}(k) = \frac{2\pi}{\hbar} \sum_{\mathbf{k_2 k_3}} \sum_{b_2 b_3, b_4} v(|\mathbf{k} - \mathbf{k_2}|)^2 \times \left(N^{\mathrm{in}} - N^{\mathrm{out}}\right) \delta(\Delta\varepsilon) \qquad \text{Supplementary Equation 2}$$

where

$$N^{\mathrm{in}} = [1 - n_{\mathrm{b}}(k)] n_{\mathrm{b}_2}(k_2) \left[1 - n_{\mathrm{b}_3}(k_3)\right] n_{\mathrm{b}_4}(|\mathbf{k} - \mathbf{k_2} + \mathbf{k_3}|) \qquad \text{Supplementary Equation 3}$$

$$N^{\text{out}} = n_{\text{b}}(k)\left[1 - n_{\text{b}_2}(k_2)\right]n_{\text{b}_3}(k_3)\left[1 - n_{\text{bb}_4}(|\mathbf{k} - \mathbf{k}_2 + \mathbf{k}_3|)\right] \qquad \text{Supplementary Equation 4}$$

$$\Delta\varepsilon = \varepsilon_{\text{b}}(k) - \varepsilon_{\text{b}_2}(k_2) + \varepsilon_{\text{b}_3}(k_3) - \varepsilon_{\text{b}_4}(|\mathbf{k} - \mathbf{k}_2 + \mathbf{k}_3|) \qquad \text{Supplementary Equation 5}$$

Here, the statically screened effectively two-dimensional Coulomb potential is

$$v(q) = \frac{1}{A}\frac{e^2}{2\varepsilon_{\text{bg}}}\frac{1}{q + \kappa} \qquad \text{Supplementary Equation 6}$$

where ε_{bg} is the background dielectric constant and κ is the inverse screening length. For simplicity, the Coulomb interaction matrix element is taken to be same for intraband and interband scattering.

II. Numerical Results:

In the spirit of our simple two-dimensional electron-gas model, we use a static inverse screening length κ that is assumed to be constant for our calculations. We estimate the value of κ from the relation[4]

$$\kappa = \frac{e^2}{2\pi\varepsilon_{\text{b}}\hbar^2}\left[m_{\text{c}}n_{\text{c}}(k = 0) + m_{\text{h}}n_{\text{h}}(k = 0)\right] \qquad \text{Supplementary Equation 7}$$

which is valid for two-dimensional systems. Here, carrier distributions at the electron and hole band bottoms $n_{\text{c}}(k = 0)$ and $n_{\text{h}}(k = 0) = 1 - n_{\text{v}}(k = 0)$ enter, and we use average values for these distribution functions of $n_{\text{c}}(k = 0) = 1 - n_{\text{v}}(k = 0) = 0.15$ as they typically occur in the computed dynamics. For the background dielectric constant we take $\varepsilon_{\text{bg}} = 19\varepsilon_0$ from Ref. 5.

We use the effective mass of the short axis of the Ti 3*d* bands as effective mass of the conduction band with m_{c} = 0.44 m_{e} and the effective mass of the Se 4*p* band for the valence band with m_{v} = -0.21 m_{v} (K. Rossnagel *et al.*, unpublished). With regard to the importance of the band anisotropy, we remark that it is likely that electron-phonon scattering helps establish radially symmetric carrier distribution[6], as we assume in our calculation with parabolic bands.

The initial condition for the dynamics is sketched in Supplementary Figure 2. Assuming a filled valence band, representing the Se 4*p* band, and an empty conduction band, which represents the Ti 3*d* band, for the unexcited system. We model the ultrafast excitation of carriers in the conduction band from lower lying states as an instantaneous process that results in a non-equilibrium distribution in the conduction band. Since the initial optical excitation originates from 1.6 eV below the Ti 3*d* band around the center of the Brillouin zone, the occupied backfolded Se 4*p* band around the $\overline{\text{M}}$-point is not affected, and we start the calculation of the evolution of the carrier density with an initially filled valence band. The initial carrier distribution in the conduction band is centered at 250 meV above E_{F} and is assumed to be of Gaussian shape in k. We numerically determine the subsequent time evolution of the carrier density for different sizes of the band gap. While the initial non-equilibrium distribution is chosen such that it qualitatively models the optical excitation, we cannot determine the excited carrier density accurately, and therefore treat this quantity strictly as parameter. To assess the dependence of our results on the excited carrier density, we compare two dynamical simulations with different excitation densities, namely the density $N_1^{\text{D}} = 2.1 \times 10^{11}$ cm^{-2} and $N_2^{\text{D}} = 2N_1^{\text{D}}$.

We next discuss some calculated results for different gap sizes. According to Monney *et al.* (see ref. 3), the gap size at liquid nitrogen temperature is about 100 meV. Upon optical excitation, the gap closes as a function of time, and we choose for our calculation three representative gap sizes of 10 meV, 50 meV and 100 meV, as shown in Supplementary Figure 3a. In particular, the valence band states are at negative energies. Supplementary Figure 3b-d show snapshots of the carrier distributions: at t = 0 fs, when the carriers are created, at t = 100 fs and at t = 200 fs for an excitation density of N_1^{D}. In Supplementary Figure 3, we show for clarity the hole distribution functions $n_{\mathrm{h}} = 1 - n_{\mathrm{v}}$. Plotting the distribution functions this way, it becomes obvious that in the course of the carrier scattering dynamics both electrons and holes are created, and the corresponding quasi-equilibrium distribution function in Supplementary Figure 3d is peaked around the gap. It is apparent that the characteristic time, with which the non-equilibrium distribution is changed, strongly depends on the gap size. At 100 fs, the low energy states of the conduction band are substantially populated for the smaller gap, whereas for the larger gap the low energy conduction band states are just starting to get populated. For t = 200 fs, the carrier distribution for the case of the small gap is already quite close to a quasi-equilibrium (Supplementary Figure 3d, grey curve), created by carrier-carrier Coulomb scattering. This type of scattering dynamics is often called carrier multiplication via impact ionization. Contrariwise, for the case of the larger gap, the carrier distribution is still far away from a quasi-equilibrium at t = 200 fs. For this gap size, carrier multiplication from the valence band is inefficient, and there is hardly any down-scattering to the band bottom from the optically excited electrons at higher energies because of energy conservation: For each electron that scatters down, *i.e.*, loses energy, its scattering partner must gain energy and be scattered away from the band bottom.

Supplementary Figure 3e-h explores the effect of the initially excited carrier density on the dynamics. Compared to the calculation in Supplementary Figure 3b-d, the carrier density is doubled. For the case of the small gap, Supplementary Figure 3e,f, there is no qualitative change of the carrier-multiplication effect. After t = 200 fs, the distribution functions are already close to equilibrium. In addition, the relaxation time is changed only slightly for different initial carrier densities, mainly because of the higher initial occupation in the states with high energy. For the larger gap, Figs. Supplementary Figure 3g,h, no qualitative difference is observed for the two initial densities, either.

The results in Supplementary Figure 3 are the raw data of the simulation. In order to model the finite energy resolution of the experiment, we calculate the number of particles at energy E broadened with the experimental resolution ΔE

$$\bar{N}(E) = \sum\nolimits_{\mathbf{k}} n(\vec{k}) g_{\Delta E}[\epsilon(\vec{k}) - E] \qquad \text{Supplementary Equation 8}$$

where $g_{\Delta \mathrm{E}}(\epsilon)$ is a Gaussian of width ΔE. Due to the simplicity of our model, we do not attempt a quantitative calculation of the measured cross section from the computed distribution functions, but analyze $\bar{N}(E)$ to explain some qualitative features of the energy distribution curves. In the main text, we plot this quantity separately for conduction and valence bands, as we cannot add the $\bar{N}(E)$ for the different bands.

For conduction electrons (n_{c} distributions with E > 0 in Supplementary Figure 3b-d), the broadened distributions $\bar{N}_{\mathrm{c}}(E)$ are shown in Fig. 3f-h in the main text, where the difference between the dynamics

for different gap sizes is obvious even with broadening. As we show in more detail below, this striking difference is explained by impact ionization becoming more pronounced for smaller gaps. We remark in passing that the direct comparison between the theoretical Fig. 3 and the experimental Fig. 4 in the main text is complicated by two factors. First, in the experimental energy distribution curves in Fig. 4 there already is a contribution from the valence band even for the unexcited system due to the limited energy resolution and a gap size below the energy resolution, as can be seen from the curve for a negative delay of 100 fs. Second, the momentum-dependent distribution functions are calculated for a parabolic band so that the influence of the Mexican-hat shaped band structure in the broadened distribution functions is not included.

For the valence band, Supplementary Figure 3b-d plots the hole distributions n_{h} for E < 0, whose dynamics clearly exhibit a similar dependence on the gap size as those of the electron band. For comparison with experiment, Fig. 3b-d shows the broadened valence electron distributions $\bar{N}_{\mathrm{v}}(E)$ corresponding to $n_{\mathrm{v}} = 1 - n_{\mathrm{h}}$, for which the only influence of the gap size is a shift of the broadened distributions $\bar{N}_{\mathrm{v}}(E)$. Even though the electron dynamics are strongly dependent on the gap size, one should therefore not expect to see a signature of the carrier multiplication in the valence band. Consequently, the above described experimental procedure to analyze the spectral weight of the backfolded Se 4*p* band yields a reliable measure of the CDW quenching.

In the following we quantify the carrier multiplication effect by determining the ratio of the time-dependent excess carrier density in the conduction band and the excited carrier density, $(N(t) - N_{\mathrm{exc}})/N_{\mathrm{exc}}$. Supplementary Figure 4a plots this quantity at t = 200 fs for gap sizes between 130 meV and 10 meV, and for the two densities discussed above. Supplementary Figure 4a clearly demonstrates that carrier multiplication is indeed pronounced for small gaps, which also speeds up the fast scattering dynamics. This is the basis for the amplification of the carrier dynamics due to the closing of the gap mentioned in the main text. For larger band gaps, above 100 meV, almost no carrier multiplication occurs.

Finally, in Supplementary Figure 4b, we plot carrier multiplication vs. time for the smaller density and a gap size of 10 meV. The computed carrier multiplication increases up to and beyond 200 fs, even though it increases more slowly at later times. This disagreement with the measurement for times longer than 200 fs, *c.f.* Fig. 2b of the main text, is to be expected, because in the experiment the gap is only partially quenched, and we have included only electron-electron scattering in our calculation. The latter is a valid approximation on very short time scales, but around 200 fs and later electron-phonon scattering also becomes important, which is likely to change the carrier multiplication dynamics, and will definitely lead to a reduction of carrier multiplication as the electron-phonon interaction cools the electronic system after several hundred femtoseconds.

Supplementary References

1. Monney, C. *et al.* Spontaneous exciton condensation in 1T-$TiSe_2$: BCS-like approach. *Physical Review B* **79,** 045116 (2009).
2. Gierz, I. *et al.* Tracking Primary Thermalization Events in Graphene with Photoemission at Extreme Time Scales. *Phys Rev Lett* **115,** 086803–5 (2015).
3. Monney, C. *et al.* Temperature-dependent photoemission on 1T-$TiSe_2$: Interpretation within the

exciton condensate phase model. *Physical Review B* **81,** 155104 (2010).
4. Haug, H. & Koch, S. W. Quantum theory of the optical and electronic properties of semiconductors. *World Scientific Publishing Co. Pte. Ltd.* (2004).
5. Li, G. *et al.* Semimetal-to-Semimetal Charge Density Wave Transition in 1T−TiSe2. *Phys Rev Lett* **99,** 027404–4 (2007).
6. Mittendorff, M. *et al.* Anisotropy of Excitation and Relaxation of Photogenerated Charge Carriers in Graphene. *Nano Lett* **14,** 1504–1507 (2014).
